# Therapeutic Applications of Dental Mesenchymal Stem Cells in Alzheimer’s Disease—A Scoping Review

**DOI:** 10.3390/dj13070288

**Published:** 2025-06-26

**Authors:** Rupali Agnihotri, Sumit Gaur

**Affiliations:** 1 Department of Periodontology, Manipal College of Dental Sciences, Manipal, Manipal Academy of Higher Education (MAHE), Udupi 576104, India; rupali.a@manipal.edu; 2 Department of Pedodontics and Preventive Dentistry, Manipal College of Dental Sciences, Manipal, Manipal Academy of Higher Education (MAHE), Udupi 576104, India

**Keywords:** Alzheimer’s disease, dental pulp stem cells, human-exfoliated deciduous teeth stem cells, periodontal ligament stem cells

## Abstract

Background/Objectives: Alzheimer’s disease (AD), a neurodegenerative condition, produces dementia and cognitive debility. Lately, preclinical models of AD have shown neuroregenerative potential of mesenchymal stem cells of dental origin (DMSC). This scoping review aims to map and synthesize the evidence on the therapeutic applications of DMSCs in AD management. Methods: This review followed the Arksey and O’Malley framework for scoping reviews and PRISMA-ScR guidelines. Scopus, Medline (Pubmed), Web of Science, and Embase databases were searched for published literature until February 2025. Data was mapped according to the type of DMSC and their therapeutic properties useful in AD management, like neuro differentiation, neuroprotection through increased neuron number and vitality, anti-neuroinflammation, mitochondrial repair, and improved cognition. Results: A total of 22 articles were included. A research gap existed, as most studies were preclinical (in vitro and animal models) with no clinical trials in humans. Furthermore, they mostly evaluated neuroregenerative properties of dental pulp stem cells (DPSC) and stem cells from human-exfoliated deciduous teeth (SHED), while Periodontal ligament stem cells (PDLSC) were least studied. All the DMSCs were neuroprotective and increased neuron number and vitality. Neurodifferentiation was reported in DPSCs and PDLSCs, while DPSCs and SHEDs showed anti-neuroinflammation, mitochondrial repair, and improved cognition in AD animal models. Conclusions: Although the DPSCs and SHEDs showed promising outcomes in preclinical models of AD, a gap exists as results have not been translated into human clinical trials. Future advances may identify plausible ways of applying the DMSCs to regain the lost neurons and re-establish a healthy brain microenvironment.

## 1. Introduction

Alzheimer’s disease (AD), a neurodegenerative disorder, is characterized by worsening dementia and cognition. It is triggered by multiple genetic and environmental factors. According to the World Health Organization, people with dementia will upsurge from the present 50 million to 82 million by 2030, and will be about 152 million by 2050 [1]. It has been postulated that the brain’s cognitive ability deteriorates for decades prior to the beginning of the clinical signs of AD. As untreated AD causes fatal complications, various new drugs and therapies have been investigated to prevent its development or improve the symptoms. Unfortunately, these drugs have failed to regenerate the injured brain cells. The United States FDA has approved medications like acetylcholinesterase inhibitors and N-methyl-D-aspartate receptor antagonists for managing cognitive debility in AD [2]. However, they provide only symptomatic relief and do not prevent disease progression. A recent medication, Aducanumab, a monoclonal antibody, improved the amyloid-β (Aβ) plaque accumulation in the AD brain but did not improve the cognitive symptoms [3]. As the prevailing drug therapies are unable to cope with the complicated pathology of AD, therapies aimed at halting or reversing AD are urgently needed. The use of brain-derived neurotrophic factors (BDNF), insulin therapy, low-level laser therapy, herbal remedies, and treatments focusing on mitochondrial calcium efflux are some of the additional treatment options available for the management of AD [4].

Since the central nervous system (CNS) has limited regenerative capacity [5], administration of external stem cells capable of differentiating into neurons or neural cells like microglia, astrocytes, and oligodendrocytes is promising for managing AD [6]. Neural stem cells were applied directly for neural differentiation in AD [7]. They secreted ‘neurotrophin’, a neuroprotectant that not only repaired the damaged cells in the brain but also slowed or reversed the cognitive decline [8]. The neural stem cells reversed the cognitive decline in animal models of the disease through secretion of BDNF [9] and nerve growth factor (NGF) [10]. Furthermore, a Phase 1 clinical trial also showed promising reversal of cognitive decline in human subjects using transplantation of NGF-secreting fibroblasts [11]. However, neural stem cell transplantation is disadvantageous due to limited cell sources, safety issues, difficult isolation, and unpredictable distribution in the hippocampus and lateral ventricles of the brain [7]. Lately, the focus has shifted towards the human mesenchymal stem cells (MSCs) for the therapeutic management of AD. Their ability to differentiate into particular cell lineages, stimulate neurogenesis, and secrete various neurogenic factors may support the restoration of neurons destroyed in AD [12]. The MSCs activated microglia, stimulated A*β* clearance, promoted autophagy, and supported neurogenesis and synaptic transmission of crucial proteins in AD models [12]. They released biologically active substances like growth factors, cytokines, enzymes, genetic material, exosomes, and microvesicles in the culture medium that helped in the treatment of neurodegenerative conditions. Various MSCs from adipose tissue, bone marrow, and umbilical cord can differentiate into neurons. They showed neuroprotection, neuroimmunomodulation and neuroregeneration at the injured nerve tissue site [13].

The oral cavity is a promising source of MSC owing to its similar embryonic origin to neural cells [14]. The dental mesenchymal tissue is also called ectomesenchyme due to its interaction with the neural crest. The oral MSCs have attracted attention due to their ease of acquisition and multilineage differentiation potential. Various stem cells from oral cavity include dental pulp stem cells (DPSC), stem cells from human-exfoliated deciduous teeth (SHED), periodontal ligament stem cells (PDLSC), bone marrow stem cells (BMSC), and stem cells from apical papilla (SCAP), dental follicle, gingiva, salivary gland, and oral mucosa [14]. The DPSCs and SHEDs originate from the dental pulp of adult and deciduous teeth, respectively, while the PDLSCs reside in the periodontal ligament. The BMSCs are present in the alveolar bone proper and the SCAP are present in the apical papilla of incompletely developed teeth, while the dental follicle stem cells are present in human third molars. Among these, the DPSCs [15,16], SHEDs [17,18], and PDLSCs [19] have been widely evaluated for their therapeutic role in AD [20]. They promoted neuroregeneration and improved the CNS defects in in vitro and animal models of AD.

As a scoping review typically includes all available evidence regardless of methodological quality or risk of bias assessment, it is well-suited for synthesizing preclinical research. It enables a researcher to gather all available evidence without limiting the analysis for studies that meet stringent quality criteria. Moreover, heterogeneous data can be explored without restricting the synthesis to a specific methodology. Owing to the early-stage, heterogeneous, and preclinical nature of research related to mesenchymal stem cells of dental origin (DMSC) in AD, a scoping review was considered as most appropriate to map the existing studies, identify gaps, and highlight areas for future research. Therefore, this scoping review aims to map the existing preclinical evidence on therapeutic applications of DMSCs in AD with the following objectives: to identify (a) the nature and type of existing evidence on the applications of DMSCs in AD management, (b) the types of DMSCs and their components applied, (c) their therapeutic properties useful in AD management, and (d) research gaps in the existing evidence related to the role of DMSCs in AD management.

## 2. Materials and Methods

### 2.1. Protocol and Registration

This review was conducted according to the Arksey and O’Malley framework for scoping reviews [21] and was reported according to the Preferred Reporting Items for Systematic Reviews and Meta-analyses extension for Scoping Review (PRISMA-ScR) [22]. The detailed checklist is provided in Table S1. A review protocol detailing the objectives, inclusion criteria, and methodology for this scoping review was registered and published on the Open Science Framework (https://doi.org/10.17605/OSF.IO/4VDSN) accessed on 20 May 2025.

The research question developed was, “What is the current evidence on the therapeutic applications of DMSCs in the management of AD?”.

### 2.2. Eligibility Criteria

In this review, all the preclinical evidence (in vitro studies, animal models, and review articles) in the English language related to the applications of DMSCs in the therapeutic management of AD and published up to February 2025 was included. For studies to be included, both DMSC therapy and AD had to be mentioned in the article. Moreover, the therapeutic effect of the DMSCs in the treatment of AD had to be reported. Studies reporting effects of MSCs other than the DMSCs, editorials, and conference presentations were excluded.

### 2.3. Sources and Search Strategy

**Figure 1:**
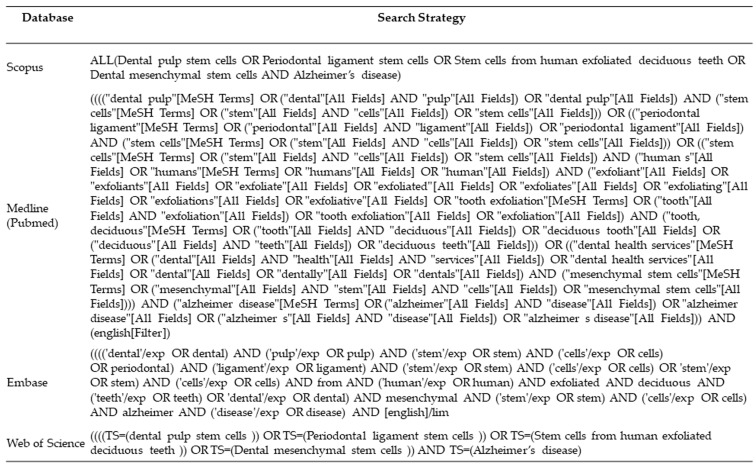
Search strategy for each database.

We applied the three-step process for searching the data as described by the Joanna Briggs Institute [23]. The initial search was conducted in online databases like Medline (PubMed), Scopus, Embase, and Web of Science up until February 2025. The titles and abstracts of the articles were analyzed to identify the relevant keywords. Combinations of keywords were then applied to identify the relevant studies. In addition, the reference list of the included articles and a hand search was performed for additional studies. The detailed search strategy for each database is given in Figure 1.

### 2.4. Study Selection

All the identified articles were screened to remove the duplicates, and two independent reviewers (RA and SG), assessed their titles and abstracts. Full texts were assessed for inclusion according to the stated objectives of the review. Any disagreements between the reviewers were resolved through discussions until an agreement was reached.

### 2.5. Data Extraction and Charting

The data was extracted and charted independently by the two reviewers (RA and SG) in a table that included authors and the year of study, study design, type of DMSC, component of DMSC and its dosage applied, the therapeutic property of the DMSC investigated, summary, and conclusion. Any differences during the process were resolved through discussions.

### 2.6. Synthesis of Results

The data was extracted and charted to summarize the results according to the type of DMSCs and their therapeutic properties useful in the management of AD.

## 3. Results

### 3.1. Selection of Sources

**Figure 2:**
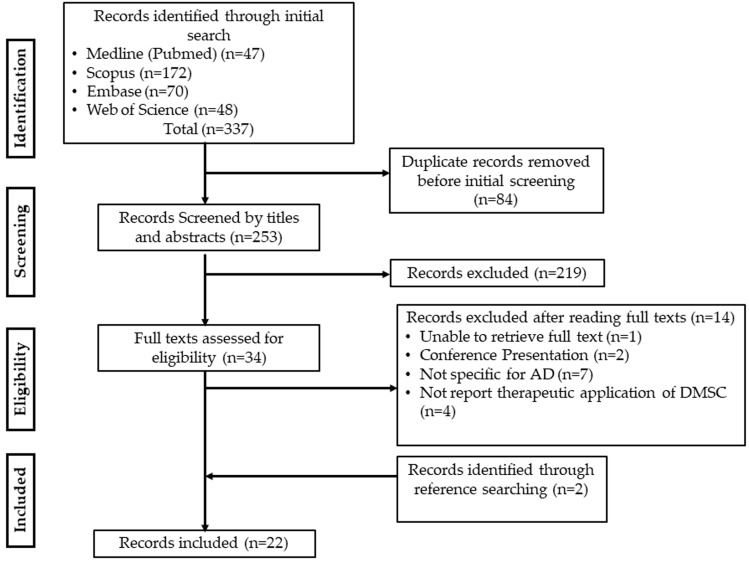
PRISMA-ScR Flow diagram.

The electronic search resulted in 337 articles [Medline (PubMed) (*n* = 47), Scopus (*n* = 172), Embase (*n* = 70), and Web of Science (*n* = 48)]. A total of 84 studies were removed due to duplication. The title and abstracts of the remaining 253 records were screened and 219 records were excluded. Subsequently, full texts of 34 articles were evaluated and 14 articles were excluded for the following reasons: unable to retrieve full text (*n* = 1), conference presentation (*n* = 2), not specific for AD (*n* = 7), and did not report therapeutic application of DMSC (*n* = 4). Two articles were identified through reference searching [24,25] resulting in a total of twenty-two articles that were included in the review. The detailed literature search process and selection procedure are depicted in a flowchart in Figure 2.

### 3.2. Characteristics of the Included Studies

**Figure 3:**
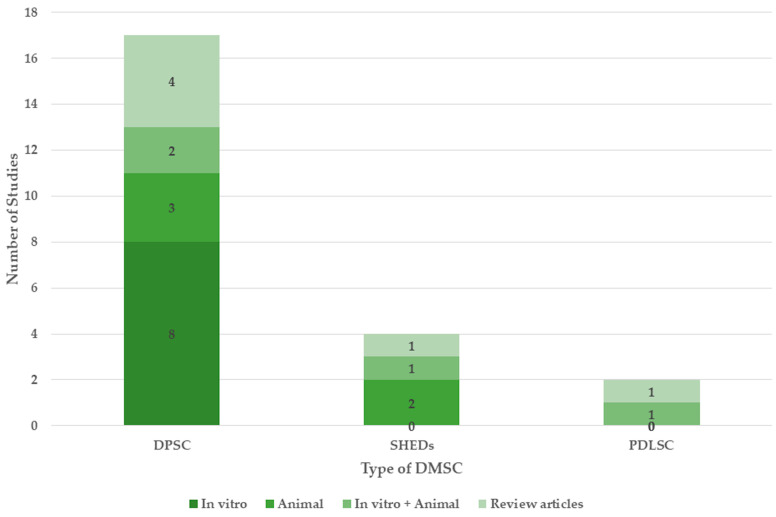
Study distribution by the type of DMSCs.

Among the 22 included studies, 8 were in vitro [15,24,26,27,28,29,30,31], 5 were animal models [16,17,25,32,33], and 4 studies utilized both in vitro and animal models [18,19,34,35], while there were 5 review articles [20,36,37,38,39] (Figure 3).

The characteristics of the included studies are detailed in Table 1. They were published between 2009 and 2025 and were mainly preclinical.

The studies applied neurotoxins like A*β* peptide (1–42) [15,24,26,32,34], okadaic acid [27], trimethylin [25], kainic acid [33], and Streptozotocin [30] in in vitro experiments and animal models to evaluate the therapeutic efficacy of DMSCs. Neural differentiation [28,29] and mitochondrial transfer [30] were mainly evaluated in in vitro studies, while neuroprotection through increased neuron number and vitality, anti-neuroinflammation, and improved cognitive functions were studied through both in vitro and animal models [15,16,17,18,19,25,31,32,33,34]. The review articles mainly discussed the role of DPSCs, SHEDs and PDLSCs in neuroregeneration through neurodifferentiation, neuroprotection, and anti-neuroinflammation [20,36,37,38,39].

### 3.3. Types of DMSCs

The different types of DMSCs investigated for their neuroregenerative properties were DPSCs [15,16,24,25,26,27,28,29,31,33,34,35], SHEDs [17,18,32], and PDLSCs [19], of which the DPSCs were the most studied cells. In vitro studies mainly utilized DPSCs and SHEDs; animal models alone or combined with in vitro models studied all the types of DMSCs. The reviews mainly focused on the neuroregenerative properties of DPSCs and SHEDs [20,36,37,38], with only one review on PDLSCs [39].

Briefly, the DPSCs and SHEDs were isolated from the pulp tissue of extracted permanent or deciduous teeth, respectively, and PDLSCs from the periodontal ligament. The tissues were enzymatically digested, and the isolated cells were cultured. The DPSCs, SHEDs, and PDLSCs were either used directly [15,16,18,19,25,27,28,31,33,34,35] or their secretomes or conditioned media (CM) [19,24,26,31,32,33], mitochondria [30] or neurospheres or spheroids [19,29] were isolated and applied in the AD model. The DMSCs were administered directly through transplantation into the hippocampus [16,25,34,35], intravenous [17,18,25], or intranasally [32]. In the included studies, the DPSCs expressed markers like- CD73, CD29, CD166, CD44, CD105, CD90, STRO-1, and vimentin, and were negative for CD14, CD19, CD31, CD34, CD38, CD45, CD133, and HLA-DR [16,24,25,27,28,34]. Embryonic stem cell markers like OCT4, Nanog, and SOX2 were also reported in two studies [28,29]. The SHEDs positively expressed CD73, CD34, CD105, CD90, and HLA-DR [17,18], while the PDLSCs were positive for CD90, CD44, CD105, and CD73 and negative for CD45 and CD34 [19]. All the DMSCs exhibited plastic adherence and spindle-shaped morphology with significantly higher proliferation and differentiation capacity than the BMSCs and ADSCs [17,18,25,27,28,29]. The differences between DPSCs and SHEDs are as given in Table 2 [40,41].

### 3.4. Therapeutic Properties of DMSCs Useful in the Treatment of AD

**Figure 4:**
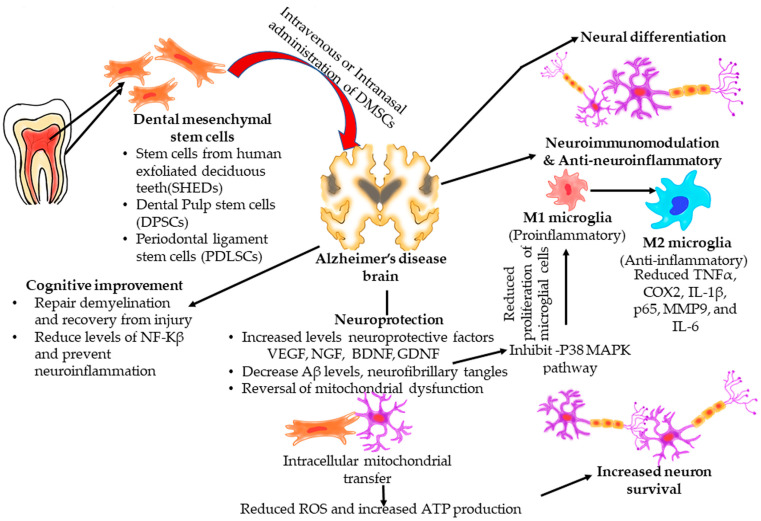
Therapeutic properties of DMSCs in AD.

The therapeutic properties of DMSCs useful in AD management are explained in Figure 4.

**Figure 5:**
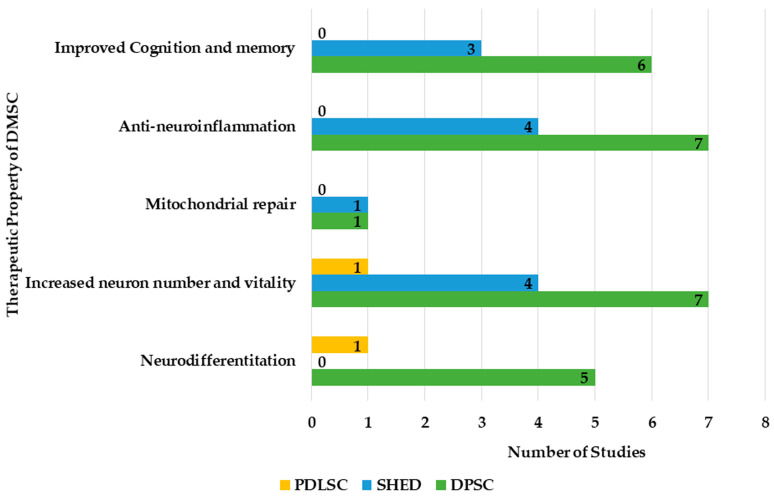
Therapeutic properties of various DMSCs.

The type of DMSCs were mapped against their therapeutic property (Figure 5).

Neurodifferentiation was reported with the DPSCs and PDLSCs. Neuroprotection by increasing the neuron number and vitality was shown by all DMSCs, while mitochondrial repair, anti-neuroinflammation, and improved cognition and memory were reported with DPSCs and SHEDs. In DPSCs, increased neuron number and vitality and anti-neuroinflammation were the most investigated properties, followed by improved cognition and memory, and neurodifferentiation. In SHEDs, the neuron number and vitality and anti-neuroinflammation were evaluated equally, while improved cognition was slightly less. In both DPSCs and SHEDs, mitochondrial repair was the least evaluated. In PDLSCs, neurodifferentiation and increased neuron number and vitality were evaluated while other properties were not investigated. Various therapeutic properties of DMSCs are detailed as follows.

#### 3.4.1. Neural Differentiation

Among the various DMSCs, the neurodifferentiation property was evaluated in DPSCs [20,28,29,35] and PDLSCs [39]. The DPSCs expressed pluripotent embryonic stemness-linked markers like OCT-4, Nanog, and Sox-2 in the presence of appropriate neuronal inducers [29]. The neuron inducers included embryonic cerebrospinal fluid (E-CSF) or growth factors and cytokines like nerve growth factor (NGF), basic fibroblastic growth factor (bFGF), Forskolin, Sonic hedgehog (SHH), and retinoic acid (RA) along with tricyclodecan-9-yl-xanthogenate (D609) [28,29]. Increased expression of immature and mature neuron markers like nestin and MAP2 was observed in human DPSCs cultured in E-CSF and RA [29]. The growth factors in E-CSF, like BMPs, IGE-2, and TGF-β3, promoted higher proliferation, neuronal differentiation, survival, and phenotypic maturation of DPSCs to adult neurons. The co-culture of human DPSCs in E-CSF containing RA resulted in a more significant percentage of viable cells [29]. Moreover, the human DPSCs showed specific neuronal morphological characteristics like bi- and multi-polar soma, large euchromatin nucleus, prominent nucleolus, and granular cytoplasm with Nissl bodies. They exhibited cytoplasmic processes and well-developed synapses with contiguous button-like areas of the cells, indicating a functional connection between the neurons [29]. The DPSCs exhibited the highest neuron-like morphologies with elevated cholinergic neuron-specific markers in the presence of bFGF, Forskolin, SHH, and RA, followed by D609, and lowest with the NGF treatment. The combined bFGF, SHH, and RA increased the expression of neuronal markers and neurotransmitters, while NGF increased reactive oxygen species (ROS), which reduced neurogenic differentiation [28]. Lately, it was shown that the DPSCs activated the Wnt8b/β-catenin signaling pathway, which enhanced the hippocampal neural regeneration [35]. Similarly, the PDLSCs differentiated into neural-like cells through a dedifferentiation stage followed by a differentiation process without cell division [39].

#### 3.4.2. Neuroprotection

All the DMSCs, either in the form of cells or secretomes showed neuroprotective effects on SH-SY5Y cells and hippocampal neurons exposed to neurotoxins. The DPSCs [15,16,24,26,27,31,33,34,35], SHEDs [17,18,32], and PDLSCs [19] were neuroprotective, as they increased the neuron number and vitality, mitochondrial repair, reduced neuroinflammation, produced neuroimmunomodulation, and inhibited neuronal apoptosis. These are explained as follows.

##### Increasing the Neuron Number and Vitality

Studies reported higher levels of factors like VEGF, RANTES, Fractalkine, FLT-3, GM-CSF, and MCP-1 in DPSCs compared to BMSC and ADSCs [26,37]. The DPSCs also expressed neuronal markers like NSE, beta-tubulin III, MAP2, GFAP, and nestin (a neuronal stem cell marker) [15]. RANTES and VEGF are neuroprotective, MCP-1 and Fractalkine are antiapoptotic, FLT-3 regulates microglial activation, while GM-CSF reverses cognitive impairment and amyloidosis [26]. The neurotrophic growth factors like VEGF, NGF, BDNF, Glial cell-derived neurotropic factors (GDNF), BMP2, bFGF, CNTF, IGF-1, FGF-2, TGF-β1, NT3, and erythropoietin expressed by DPSCs supported neuron survival [15,24,33,37]. The BDNF, NGF, and GDNF induced endogenous cell differentiation in impaired neuronal cells and stimulated them to secrete other neurotrophic factors [15]. They also increased neuroprotective cytokines like IL-5, IL-6, IL-8, and IL-10, as well as chemokines like MCP1 and RANTES [24]. DPSC secretomes increased neprilysin, a membrane-bound protease that effectively degraded the neurotoxin A*β* peptide [26]. Subsequently, the SH-SY5Y cells or hippocampal neurons exposed to A*β*1–42 and treated with DPSC or secretome (5 μg/mL) retained their cellular morphology, including the cytoplasm, the cell margins with elongated dendrites and increased cell density and viability, and reduced apoptosis compared to untreated cells [15,24,26]. The DPSC secretome reduced cytoskeletal damage in SH-SY5Y cells, which was indicated by elongated dendritic bundles of thick microtubules and increased growth factors that antagonize tau phosphorylation [27]. Moreover, intrahippocampal transplantation of DPSCS increased neuron-related doublecortin, Neuronal nuclei (NeuN), and neurofilament (NF)-200 expression in the hippocampus and decreased the Aβ levels, further strengthening their neuroprotective ability [16,33].

The DPSC secretomes protected neuronal cells from apoptosis and attenuated oxidative stress due to higher VEGF, FGF2, and IL-6 levels that scavenge superoxide radicals. They stimulated endogenous survival factor Bcl-2 and decreased the apoptotic regulator Bax which reduced apoptosis of cells exposed to A*β* peptide [24,26,34,35]. The increased activity of antioxidant enzymes like catalase, glutathione-s-transferase, glutathione-peroxidase, and superoxide-dismutase further prevented neuronal apoptosis [24].

Likewise, SHEDs injected in the hippocampus reduced the levels of phosphorylated tau responsible for neurodegeneration and cognitive impairment. They increased the number of neurons in the brain and restored their function. Although SHEDs did not replace the damaged cells by differentiating into neurons, they secreted the nutrient factors and promoted cell regeneration in the damaged part [17]. The SHED-CM suppressed glutamate-induced neuronal death and significantly increased the cell viability [32].

Similarly, secretome from the 3D cultured PDLSC spheroids treated with brain homogenate of AD contained neuron regenerating factors [19]. The treated PDLSCs expressed three proteins, including PTPN6 or SHP-1, muscle PYGM, and FAM90A20P, related to signaling for CNS development, neuron differentiation, astrocytes and oligodendrocytes development, neurotransmission, axon regeneration, and inhibition of neuroinflammation.

##### Mitochondrial Repair

As mitochondrial dysfunction causes neuronal cell death [42], internalization of mitochondria isolated from DPSCs by neural cells was promising in treating AD [30]. As mitochondria spontaneously translocate amid the cells during tissue homeostasis, mitochondria from healthy stem cells can restore the nonfunctional mitochondria in damaged cells. The isolated mitochondria co-incubated with mammalian cells were spontaneously internalized as autonomous mitochondria and promoted neuronal survival and metabolism. Their neuroprotective effect occurred at a concentration of 40 μg/mL, wherein they reduced ROS generation, promoted neurite extension and retention, reversed neurite damage, and reduced tau and amyloid accumulation. There was no cellular toxicity, and increased ATP production protected the neurons [30].

Likewise, SHED treatment increased ATP production and expression level of Hook3, a protein that affects the axonal transport in neurons [18]. SHEDs repaired the mitochondrial axonal transport and increased the neurons’ ATP production and mitochondrial membrane potential. They reduced tau phosphorylation and increased the normal tau, leading to increased levels of nicotinamide adenine dinucleotide family proteins and ATP in neurons [18].

##### Anti-Neuroinflammation and Neuroimmunomodulation

The culture supernatant of DPSCs had antioxidant, anti-neuroinflammatory, and neurotrophic properties. It suppressed the total intracellular and mitochondrial-specific ROS in microglia. The BDNF and GDNF in the CM blocked the phosphorylation site (tyr 182) of P38 and reduced microglial cell proliferation via the P38 MAPK pathway [31]. The DPSCs not only reduced the lipopolysaccharide (LPS)-induced neuroinflammation and ROS production, but also increased the antioxidant levels. They significantly suppressed the downregulation of antioxidant factors like Nrf2, HO-1, GPX4, and SOD1 through upregulation of the AKT/GSK3β pathway in the in vitro cellular model of AD [34]. Subsequently, the levels of Nrf2 increased in the nucleus and reduced oxidative stress and neuroinflammation. The pro-inflammatory markers like TNFα, COX2, IL-1β, p65, MMP9, and IL-6 were reduced with increased levels of anti-inflammatory markers such as IL-10, IL-4R, Arg-1, and alpha 2 macroglobulin in the presence of DPSCs [31,34,36]. Likewise, the SHED -CM increased the anti-inflammatory factors TNF-β and IL-6, whereas the pro-inflammatory factors CXCL-1, IL-1, IL-5, interferon gamma receptor 1, IL-2, and IL-4 were greatly decreased [18]. The alpha-2 macroglobulin could bind the misfolded proteins and promote Aβ clearance [36]. The DPSCs reduced the nuclear factor kappa β levels in the serum of rats treated with neurotoxin, indicative of their anti-neuroinflammatory potential [25]. Furthermore, it reduced reactive astrocytes and IBA-1 expression in the inflamed hippocampus [33].

The DPSCs mitigated oxidative stress and neuroinflammation caused by microglial cells [31]. The oxidative stress induced mitochondrial dysfunction in the microglia. However, the DPSCs supernatant reduced the mitochondrial membrane potential, leading to reduced energy production and metabolic activity in microglia. Subsequently, it led to cell cycle arrest at the G0/G1 phase and decreased the proliferation of microglia.

The DPSCs and the SHED-CM converted the pro-inflammatory brain environment into anti-inflammatory by modifying the phenotype of the microglia [17,32,34]. The SHED -CM reduced microglial activation as indicated by decreasing the expression of IBA-1 (a marker of activated microglia) [17]. CM from both DPSCs and SHEDs shifted the M1-type pro-inflammatory microenvironment associated with AD toward the M2-type anti-inflammatory/neuroprotective one [32,34]. Specifically, the SHED-CM contained M2 inducers like monocyte chemoattractant protein-1 [MCP-1] and secreted ectodomain of sialic acid-binding Ig-like lectin-9 [sSiglec-9]. They could switch the M1 to M2 phenotype and promote nerve regeneration, leading to neuronal plasticity and neurogenesis in AD [36].

#### 3.4.3. Positive Effect on Cognitive Function and Memory

The intrahippocampal injections of neurotoxins Aβ and TMT induced dementia and cognitive dysfunction in various animal models [16,25,32,33]. The Aβ decreased choline acetyltransferase activity in the medial septum, cortex, and hippocampus, and TMT damaged the hippocampal neurons, caused pyramidal cell loss in the CA1 area in a dose-dependent manner, and suppressed the hippocampus neurogenesis [25]. The DPSCs can differentiate into cholinergic neurons and express choline acetyltransferase, which may support cognitive function [28,38].

The intrahippocampal transplantation of DPSCs improved behavioral and cognitive function in Aβ-induced AD [16,25,33,34,35]. As they were injected into the hippocampus, the cells directly reached the injury site and regulated the secretion of neuron-related proteins and reduced the time required to complete the memory tasks [16]. The DPSCs repaired demyelination and promoted recovery from peripheral nerve injury. They significantly reduced the percentage of damaged CA1 pyramidal neurons and also reduced the NF-κβ levels, and improved the passive avoidance memory in rats [25]. The pathological Aβ aggregation and the expression of phosphorylated tau protein was much less in the hippocampus of human DPSCs-treated AD mice compared to the controls, which improved the cognitive function [34,35]. The human DPSCs had a positive impact on the spatial learning and memory function in AD mice models [34,35].

SHED transplantation also decreased cognitive impairment by inhibiting apoptosis of neurons in the hippocampus of the CA1 region [32]. They reduced amyloid plaque deposition and tau phosphorylation, which deposit A oligomers and induce the release of neurotoxic mediators from M1-type microglia, resulting in neuronal death and reduced neurotransmission. However, the M2-cells suppress M1-related inflammation and increase BDNF-encoding mRNA, which supports synaptic remodeling in the adult hippocampus and improves memory. The SHED -CM induced anti-inflammatory M2-like microglia, attenuated the pro-inflammatory responses, and increased the anti-inflammatory factors, creating an environment conducive to neuroregeneration [32]. As a result, axonal elongation and improved neurotransmission were observed. Furthermore, the SHED-CM protected cerebral neurons from glutamate neurotoxicity and improved cognition more effectively than the CM from other stem cells like bone marrow or fibroblasts [32]. It upregulated the PPARγ signaling pathway and improved cognition by increasing glucose metabolism and reducing Aβ deposition in SAMP-8 mice brain [17]. They also recovered dysfunctional mitochondria, leading to improved cognition [18].

## 4. Discussion

The present scoping review aimed to map the therapeutic applications of DMSCs in AD management. Overall, the results from the preclinical studies showed a positive impact of DMSCs, specifically the DPSCs and SHEDs in AD. However, the included research was mainly in vitro and animal studies, and more clinical studies in humans with AD are needed to evaluate the impact of DMSCs. Unclear pathophysiology of AD and neuroregenerative mechanisms of DMSCs could be one of reasons limiting their applications in vivo [4].

In general, the DMSCs exhibit typical phenotypic properties of MSCs like fibroblast-like shape, expression of specific markers like CD73, CD44, CD51, CD105, CD29, CD90, and STRO-1, and absence of unique hematopoietic stem cell markers like CD34, CD79a, CD11b, CD45, CD14, CD19, and HLA-DR. Moreover, they can differentiate into chondrocytes, osteocytes, and adipocytes. Specifically, they can transdifferentiate into mesenchymal neuronal and epithelial cells [43]. Even though various DMSCs like DPSCs, SHEDs, and PDLSCs are derived from the same source, their biological and functional properties are different due to niche influence. They express specific MSC markers and phenotypic characteristics. Their easy of accessibility, high proliferation rate multilineage differentiation, immunomodulatory properties, and neural crest origin have increased their acceptability for neuroregeneration over other non-DMSCs.

The DMSCs can either be isolated directly or through biobanking, wherein the retrieved cells are stored for later use, enabling patient-specific treatments [44]. They can be easily obtained from healthy adult teeth removed for orthodontic reasons, the wisdom teeth, or naturally exfoliated deciduous teeth. These teeth can be stored in the stem cell banks and the DPSCs and SHEDs isolated from the extirpated pulp tissue can be cryogenically preserved for future applications. The dentists should be made aware of the tooth banks that store the extracted adult teeth or naturally exfoliated teeth. The DPSCs and SHEDs may be regarded as “Bio-insurance” for the future, providing the possibility of using patient’s own cells for potential medical treatment, including that for AD if needed [45].

Owing to their versatile differentiation, the DPSCs and SHEDs have been used in regenerative endodontics to repair damaged pulp tissue and dentin after root canal therapy or injury to the pulp–dentin complex [46,47]. The DPSCs and their extracellular vesicles enhanced cellular activities and exhibited potential angiogenic capabilities, facilitating stem cell recruitment into the root canal, subsequently leading to cell differentiation [48]. A randomized controlled clinical trial was performed in patients with pulpal necrosis following traumatic tooth injury. It was observed that three-dimensional pulp tissue along with blood vessels and sensory nerves regenerated twelve months after SHEDs transplantation [49]. The PDLSCs have also shown potential for periodontal tissue regeneration owing to their self-renewal, multipotency, and immunomodulatory properties [50]. Accordingly, the PDLSCs transferred amniotic membrane and when transplanted in a rat periodontal defect model, they enhanced periodontal tissue regeneration four weeks after transplantation [51].

The DPSCs showed neural differentiation in the included studies because all DMSCs originate from the ectomesenchyme neural crest and can transform into neuron-like cells when exposed to favorable microenvironments [20,52]. When exposed to specific neuronal inducers and growth factors, the DPSCs expressed immature and mature neuronal markers. They even showed increased proliferation, neuronal differentiation, survival, and phenotypic maturation. Additionally, DPSCs, SHEDs, and PDLSCs were all neuroprotective as they increased the neuron number and vitality. The DPSCs and SHEDs reduced neuronal inflammation, promoted mitochondrial repair, and improved cognitive and memory functions. Both transplanted cells and secretomes of DMSCs were utilized in the included studies for neuroregenerative effects. The cell secretomes are the growth and trophic factors shed from the surface of the MSCs in an extracellular environment. They include both CM and extracellular vesicle fraction. The MSC secretome consists of soluble proteins and a vesicular fraction that include microvesicles and exosomes which promote transfer of proteins and genetic materials. Likewise, the CM from the DPSCs and SHEDs showed greater neurotrophic, angiogenic, anti-apoptotic, neurite outgrowth, and immunomodulatory effects due to their multidirectional paracrine effects [37].

However, these therapeutic properties were more frequently evaluated in DPSCs and SHEDs compared to the PDLSCs, which could be related to their ease of isolation and culture. The dental pulp tissue is significantly rich in stem cells and can be easily isolated from third molars or premolars extracted for orthodontic reasons. There is a key gap in the evaluation of the neuroregenerativity of PDLSCs in AD. Moreover, the dosage of the cells or their secretomes and their routes of administration were not standardized in the included studies and should be determined through further clinical trials.

In animal models, various routes like intravenous, intrahippocampal, and intranasal were applied for administration of DPSCs and SHEDs. The intravenous route is convenient, as stem cells can be delivered repeatedly through peripheral veins. However, its disadvantages include entry of the stem cells into the blood circulation and infiltration into various organs. When injected through the caudal vein, they may take time in crossing the blood–brain barrier (BBB) and to enter the hippocampus for functional activities. The intrahippocampal route avoids the BBB but requires a three-dimensional positioning device and imaging system. In addition, stereotactic injection is a traumatic operation that reaches the functional area of the hippocampus, so multiple injections cannot be performed, limiting its clinical application [53]. However, the intranasal route is preferred, as it targets the therapeutic molecules directly to the CNS along the olfactory, trigeminal, neural, and vascular routes [54]. Moreover, it is minimally invasive, easily repeatable, and a simple procedure. Furthermore, in the intranasal route, the therapeutic cells bypassed the BBB and achieved a high concentration in CNS [55]. Compared to intravenous routes, intranasal administration of therapeutic molecules improved the cognitive function significantly [56].

It is also difficult to evaluate the therapeutic potential of DMSCs in different stages of AD through preclinical studies. Large-scale clinical trials with DMSCs are also difficult due to complicated techniques of isolation, expansion, and cultures supplemented with growth factors [57]. Moreover, the potential adverse effects of this therapy in AD are unknown [58]. Ethical issues, technique sensitivity like immunogenicity, and limited cell survival in vivo are other potential limitations of DMSC therapy. In addition, the treatment with DMSCs would be expensive and difficult to translate into clinical trials. Additionally, individualization hinders the translation of pre-clinical research into clinical settings [4,59]. There may be differences when extrapolating the outcomes of animal models to humans because the human brain is far more complicated than the animal brain. The use of existing models to determine the best course of treatment is limited, since they usually ignore early-stage human diseases and other important aspects of AD [60].

The outcomes of this review are limited since they are based on preclinical in vitro animal studies and reviews. Nevertheless, they are positive, as they indicate that DMSCs may be able to reverse the neurodegeneration and cognitive decline associated with AD by modifying neural differentiation, neuroinflammation, and neuronal apoptosis, as well as increasing amyloid plaque degradation while also inhibiting tau protein hyperphosphorylation. These findings are still preliminary, and more rapid translational research in humans is required.

## 5. Conclusions

AD is one of the significant health afflictions affecting a greater proportion of the world population. Even though many hypotheses have been given for its pathophysiology, the treatment is only symptomatic and cannot reverse the disease process. As AD has a complex pathophysiology, multiple treatment modalities may be required to treat the various spectrums of this condition. DMSC therapy is a promising approach to the therapeutic management of AD. DMSCs promote neurogenesis, anti-neuroinflammation, neuroimmunomodulation, and neuroprotection. DMSCs like DPSCs and SHED have shown promising results in preclinical and animal models, which are difficult to translate into human clinical trials. However, advances in medical tissue engineering may help identify plausible ways of applying DMSCs to regain the lost neurons and restore the healthy brain microenvironment in AD.

## Figures and Tables

**Table 1:** Characteristics of the included studies.

Author	Study Type	Type of DMSC	Component of DMSC Applied and Dosage	Therapeutic Property of DMSC Applied	Summary and Conclusion
Apel C. et al., 2009 [15]	In vitro	DPSC	Cells 5 × 10^4^ cells per insert	Neuroprotection: Increased neuron number and vitality	DPSCs mitigated the impact of neurotoxins, exhibited a neuronal phenotype, generated neurotrophic effects, and safeguarded primary neurons in in vitro models of Alzheimer’s disease and Parkinson’s disease.
Yalvac M.E. et al., 2013 [24]	In vitro	DPSC	Secretome 1:5 ratio of Secretome SH-SY5Y cells	Neuroprotection Increased neuron number and vitality	DPSCs increased antioxidant enzyme activity and reduced neuronal apoptosis.
Mita T. et al., 2015 [32]	Animal model	SHED	Conditioned medium 50 μL	Neuroprotection Increased neuron number and vitality Anti-neuroinflammation Improved Cognition and memory	Intranasal administration of SHEDs substantially improved cognitive function and their conditioned medium produced a tissue-regenerating environment.
Ahmed N.E. -M.B. et al., 2016 [26]	In vitro	DPSC	Secretome 5 μg/mL	Neuroprotection Increased neuron number and vitality	The DPSC secretome had the highest concentrations of growth factors, decreased the apoptotic regulator Bax, enhanced neuronal cell viability, raised the endogenous survival factor Bcl-2, and substantially lowered the cytotoxicity of A*β* peptide. Within 12 h, the neprilysin enzyme in DPSC secretome degraded Aβ1-42.
Wang F. et al., 2017 [27]	In vitro	DPSC	Cells 6 × 10^4^ cells	Neuroprotection Increased neuron number and vitality	DPSCs facilitated neuroregeneration, as seen by elongated dendrites, densely organized microfilaments, thickened microtubular fibrils, enhanced cell survival, reduced apoptosis, and tau phosphorylation.
Kang Y.H. et al., 2019 [28]	In vitro	DPSC	Cells	Neuro differentiation	DPSCs effectively trans-differentiated across all treatments and displayed neuron-like morphologies with elevated cholinergic neuron-specific markers.
Man R.C. et al., 2019 [36]	Review	DPSC SHED	Secretome	Neuroprotection Increased neuron number and vitality Anti-neuroinflammation	Alpha 2 macroglobulin derived from DSCs secretome bound the β-amyloid plaque and promoted its clearance while fractalkine promoted phagocytic functions. Siglec-9 and MCP-1 promoted nerve regeneration by converting M1 to M2 phenotype.
Goudarzi G. et al., 2020 [29]	In vitro	DPSC	Neurospheres	Neuro differentiation	Human hDPSCs treated with 5% embryonic cerebrospinal fluid and cultured in media containing DMEM, retinoic acid, glial-derived neurotrophic factor and brain-derived neurotrophic factor showed neuron like features.
Zhang X.M. et al., 2021 [16]	Animal Model	DPSC	Cells 5 × 10^6^ cells	Neuroprotection Increased neuron number and vitality Improved Cognition and memory	DPSCs enhanced cognitive and behavioral functions by upregulating the expression of neuron-related doublecortin, NeuN, and neurofilament 200 while downregulating amyloid-β.
Bar J.K. et al., 2021 [37]	Review	DPSC	Secretome	Neuroprotective Angiogenesis Neuronal growth	DPSC secretome through paracrine mechanism enhances neuronal survival and reduces apoptosis through RANTES, FGF2, and Fractalkine, supports neuronal growth through BDNF and NGF and angiogenesis through VEGF.
Malekzadeh S. et al., 2022 [25]	Animal Model	DPSC	Cells 1 × 10^6^ cells/mL	Neuroprotection Anti-neuroinflammation Improved Cognition and memory	Transplanting DPSCs enhanced learning and memory while lowering the percentage of injured pyramidal neurons and the NF-Kβ serum level.
Venugopal C. et al., 2022 [33]	Animal Model	DPSC	Cells/ Secretome 4 μL of cells or Secretome	Neuroprotection Increased neuron number and vitality Anti-neuroinflammation Improved Cognition and memory	In addition to preventing neurodegeneration and neuroinflammation, DPSCs also improved neurogenesis, spatial learning, and memory, decreased pro-apoptotic factors, increased anti-apoptotic factors, and raised the expression of endogenous neural survival factors. Results were better with DPSCs and their secretome than with bone marrow-derived stem cells and their secretome.
Zhang X. et al., 2022 [17]	Animal Model	SHED	Cells 5 × 10^5^ cells	Neuroprotection Increased neuron number and vitality Anti-neuroinflammation Improved Cognition and memory	Transplanted SHED entered the brain and improved the glucose metabolism in AD mice by upregulating the PPARγ signaling pathway
Guo W et al., 2022 [18]	In vitro Animal Model	SHED	Cells 2 × 10^6^ cells	Neuroprotection Increased neuron number and vitality Mitochondrial repair Anti-neuroinflammation Improved Cognition and memory	Treatment with SHED reduced AD symptoms, enhanced cognitive performance, and restored memory loss in SAMP8 mice, potentially by restoring damaged mitochondria through the mitochondrial pathway, Hook3, Mic13, and MIF.
Mohebichamkhorami F. et al., 2022 [19]	In vitro Animal Model	PDLSC	Secretome Spheroids 20 mg/mL	Neuroprotection Increased neuron number and vitality	Modified secretome of 3D cultured spheroids of PDLSCs treated with BH-AD was a reservoir of regenerating neural factors useful in AD treatment.
Dong Z. et al., 2022 [38]	Review	Oral MSC (DPSC)	-	Neural differentiation Neuroprotection Anti-inflammation	Reduced symptoms of AD and improved cognitive function.
Mohebichamkhorami F. et al., 2022 [39]	Review	PDLSC	-	Neural differentiation	After a phase of dedifferentiation, PDLSCs undergo a differentiation process without cell division to become neural-like cells.
Xiong W. et al., 2022 [20]	Review	DPSC	-	Neural differentiation Neuroprotection Neuronal regeneration Anti-neuroinflammation	DPSCs are an important source of stem cells for the regeneration of neurons or protection of existing neurons in the neurodegenerative diseases like AD
Mishra M. et al., 2023 [30]	In vitro	DPSC	Mitochondria 40 g/mL	Neuroprotection Mitochondrial repair	Internalization of DPSC-derived mitochondria produced significant neuroprotection in the cellular AD.
Howlader M.S.I. et al., 2024 [31]	In vitro	DPSC	Secretome 2 mL	Neuroprotection Anti-neuroinflammation	DPSC secretome decreased inflammatory markers, induced anti-inflammatory molecules in microglial cells, and decreased mitochondrial membrane potential in microglial cells. Subsequently, microglial cell proliferation was inhibited, the MAPK P38 pathway and downstream signaling of inflammation were inhibited, intracellular ROS and their production from mitochondria were decreased.
Xiong W. et al., 2024 [34]	In vitro Animal model	DPSC	Cells	Neuroprotection Anti-neuroinflammation Improved Cognition and memory	In in vitro AD models, human DPSCs regulated the polarization of hyperactive microglia cells, decreased oxidative stress, and encouraged neuronal repair. Nrf2 nuclear accumulation and the production of downstream antioxidant enzymes via the AKT-GSK3β-Nrf2 signaling pathway were the mechanisms underlying these effects.
Xiong W. et al., 2025 [35]	In vitro Animal model	DPSC	Cells	Neuronal differentiation Improved Cognition and memory	Human DPSCs activated the Wnt/β-catenin pathway which stabilized the hippocampal neural network and reversed memory deficits and promoted neural regeneration.

**Table 2:** Difference between DPSCs and SHEDs.

	DPSCs	SHEDs
Origin	Adult human dental pulp of impacted third molars, orthodontic teeth and supernumerary teeth	Exfoliated human deciduous teeth
Characteristics	Increased clonogenicity, proliferation and ability to form mineralized nodules	Increased proliferation
Differentiation potential	Potential for multilineage differentiation; ability to differentiate into neural cells, endothelial cells, myocytes, hepatocytes, adipocytes, chondrocytes, osteoblasts, odontoblasts, and pancreatic cells. Additional differentiation into cardiomyocytes and corneal epithelial cells	Differentiate into osteoblasts, odontoblasts, adipocytes, chondrocytes, neural cells, endothelial cells, myocytes, hepatocytes, and pancreatic cells
Proliferation rate	Lower proliferation rate than SHEDs	Exhibits a superior proliferation rate compared to DPSCs and BMSCs, attributed to enhanced expression of genes associated with cell proliferation, such as fibroblast growth factor-2 and transforming growth factor-β, and elevated expression of stemness markers.
Stem cell Markers	MSC markers: CD29, CD44, CD90, CD166, STRO-1, and CD146	MSC markers: CD73, CD13, CD90, CD105, CD166, and STRO-1 Embryonic stemness markers: OCT-4 SOX-2 and NANOG

## Data Availability

No new data were created or analyzed in this study. Data sharing is not applicable to this article.

## Supplementary Materials

The following supporting information can be downloaded at: https://www.mdpi.com/article/10.3390/dj13070288/s1, Table S1: Preferred Reporting Items for Systematic reviews and Meta-Analyses extension for Scoping Reviews (PRISMA-ScR) Checklist. Reference [22] are cited in the supplementary materials.

## Abbreviations

The following abbreviations are used in this manuscript:

AD	Alzheimer’s disease
MSC	Mesenchymal stem cells
DMSC	Mesenchymal stem cells of dental origin
DPSC	Dental pulp stem cells
SHED	Human-exfoliated deciduous teeth
PDLSC	Periodontal ligament stem cells
BMSC	Bone marrow stem cells
CNS	Central nervous system
ROS	Reactive oxygen species
NFT	Neurofibrillary Tangles
CM	Conditioned media
NGF	Nerve growth factor
bFGF	Basic fibroblastic growth factor
SHH	Sonic hedgehog
RA	Retinoic acid
D609	Tricyclodecan-9-yl-xanthogenate
NeuN	Neuronal nuclei
BDNF	Brain-derived neurotrophic factor
GDNF	Glial cell-derived neurotropic factors
NF	Neurofilament
